# Postoperative Cauda Equina Syndrome Revealing a Delayed-Onset Dural Tear Following Lumbar Spine Surgery: A Case Report

**DOI:** 10.7759/cureus.89467

**Published:** 2025-08-06

**Authors:** Safumi Ogawa, Yohei Inomata, Kazuki Yamao, Shogo Karino, Kazuyuki Watanabe

**Affiliations:** 1 Department of Orthopedic Surgery, Iwaki City Medical Center, Iwaki, JPN; 2 Department of Research for Spine and Spinal Surgery, Fukushima Medical University, Fukushima, JPN; 3 Department of Orthopedic Surgery, Fukushima Medical University, Fukushima, JPN

**Keywords:** cauda equina syndrome, cerebrospinal fluid leakage, delayed dural tear, lumbar spinal stenosis (lss), lumbar spine surgery

## Abstract

Dural tears are a well-known complication of spinal surgery. While most occur intraoperatively and are promptly identified, some are overlooked or develop postoperatively. Delayed-onset dural tears are relatively rare but can result in significant neurological complications, including cauda equina syndrome (CES). We report the case of a 74-year-old woman who developed delayed-onset CES following lumbar decompression surgery. Postoperatively, the patient experienced saddle anesthesia and urinary retention. MRI revealed a hematoma compressing the cauda equina. Emergency reoperation revealed a longitudinal dural tear with herniation and incarceration of the cauda equina. The nerve roots were successfully reduced, and the dural defect was repaired. The patient showed partial neurological recovery postoperatively. In this case, intraoperative dural vulnerability, suction drainage, and increased intrathecal pressure due to straining likely contributed to delayed dural rupture and nerve root herniation. Awareness of this rare but serious complication is essential. MRI findings and clinical symptoms should prompt urgent reoperation if CES is suspected. This rare case of delayed-onset CES due to a dural tear after lumbar surgery was likely precipitated by straining-induced intrathecal pressure. The case underscores the importance of meticulous intraoperative dural inspection, cautious drainage management, and early recognition of postoperative neurological deterioration.

## Introduction

Dural tears are a recognized complication of spinal surgery, with an incidence ranging from 1% to 17% [1-4]. Although most are detected intraoperatively, some may go unnoticed or develop postoperatively. These lesions, which are not apparent during surgery but manifest later, are referred to as delayed-onset dural tears. The incidence of postoperative delayed-onset dural tears has been reported to range from 0.6 to 8.3 per 1,000 surgeries [1-4]. Complications can include delayed wound healing, surgical site infections, soft tissue swelling, persistent postural headaches, dural cysts, subdural hematomas, and adhesive arachnoiditis [1,5,6]. Associations with systemic complications such as pneumonia, urinary tract infections, thrombosis, acute kidney injury, pulmonary emphysema, and pneumothorax have also been documented [1,5,6]. Cauda equina syndrome (CES) is a severe neurological condition resulting from compression of the lumbosacral nerve roots, typically presenting with lower extremity weakness, saddle anesthesia, and bladder or bowel dysfunction. Although bladder and bowel dysfunction are associated with dural tears [7], CES resulting from delayed-onset dural tears is extremely rare. Prompt recognition and management of dural tear is recommended to prevent these complications. However, the management of delayed-onset postoperative dural tears remains poorly defined due to the rarity of such cases.

Here, we report a case of a delayed-onset dural tear following lumbar spine surgery, which resulted in CES.

## Case presentation

A 74-year-old woman presented with a two-year history of buttock pain, bilateral posterior thigh pain, and numbness in the soles of her feet. She had been diagnosed with lumbar spinal stenosis (LSS) at another hospital 1 year prior, based on MRI findings. Despite conservative treatment, her symptoms worsened, particularly during ambulation, and she was referred to our hospital. Her medical history included hypertension, dyslipidemia, and cataracts.

On presentation, she had numbness in both soles at rest, and intermittent claudication worsened her leg pain after walking approximately 200 m. No bladder or rectal dysfunction or perineal symptoms were present. Physical examination revealed lower limb numbness with lumbar extension, but Kemp’s sign was negative bilaterally. Straight leg raising and femoral nerve stretch tests were also negative bilaterally. Deep tendon reflexes were normal at the patella but absent in both Achilles tendons. Sensory examination showed hyperesthesia extending from the anterolateral thigh to the posterolateral lower legs and soles. Manual muscle testing (MMT) revealed grade 4 weakness in the bilateral gastrocnemius and flexor digitorum longus.

**Figure 1:**
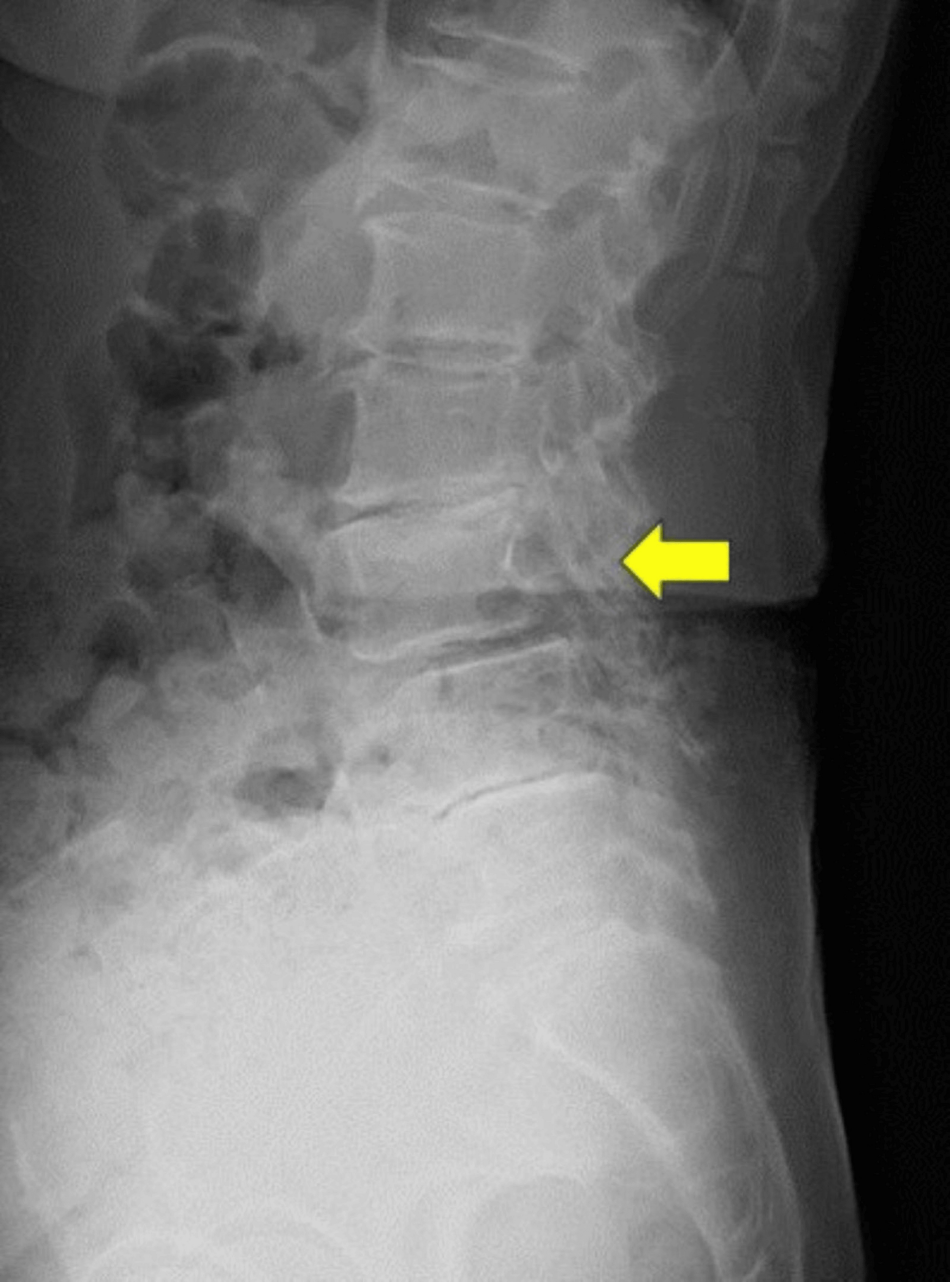
Preoperative lateral lumbar radiograph. Lumbar radiograph at initial presentation showing anterior slippage of L3 (yellow arrow).

**Figure 2:**
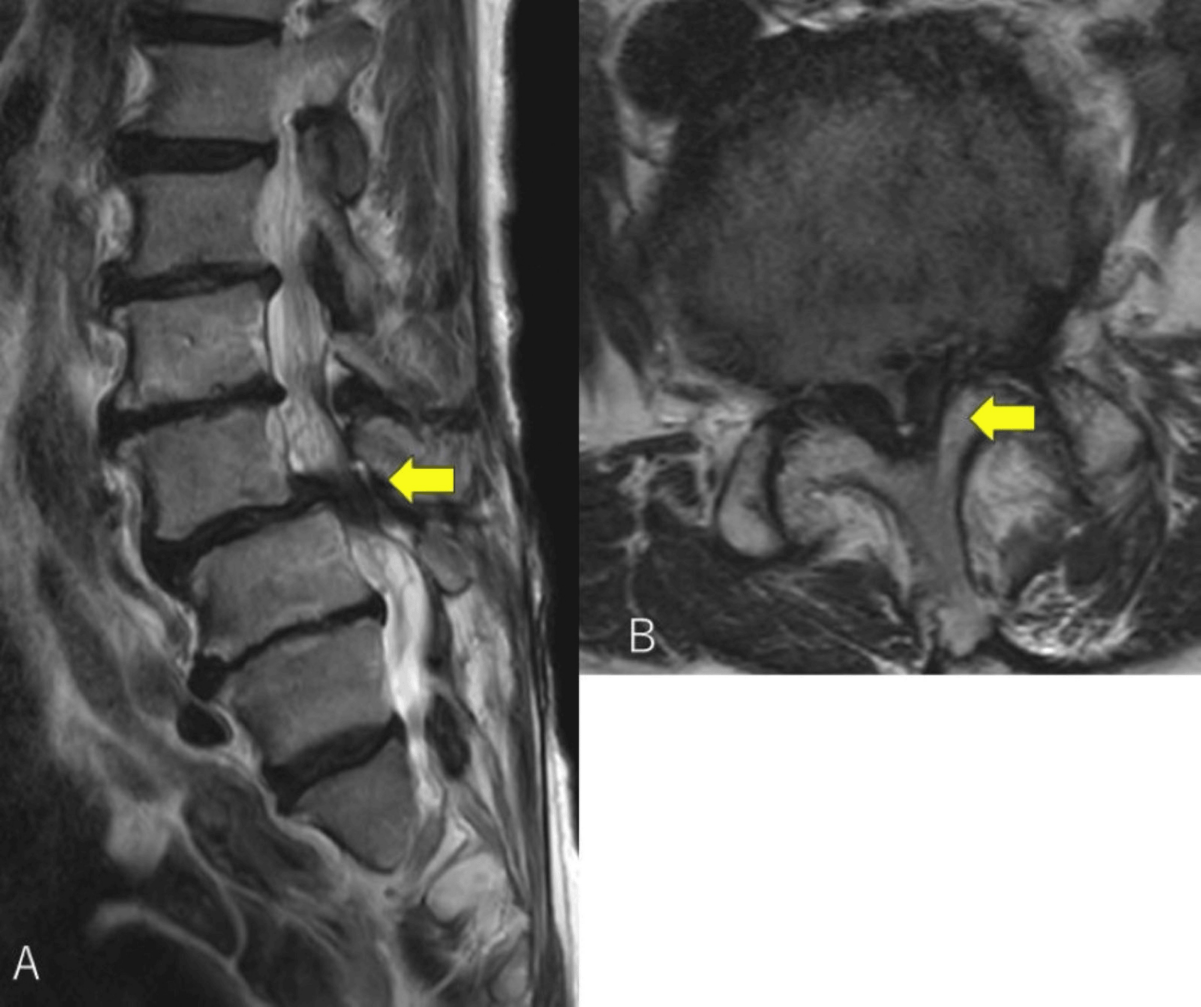
Preoperative lumbar MRI. Preoperative lumbar MRI demonstrating L3 slippage and lumbar spinal stenosis at the L3-4 level (yellow arrow) on the sagittal plane (A), and severe stenosis at L3-4 on the axial plane (B).

Plain lumbar radiographs showed anterior slippage of L3 (Figure 1), and lumbar magnetic resonance imaging (MRI) revealed severe canal stenosis at L3-4 (Figures 2A, 2B). She was diagnosed with mixed-type LSS secondary to L3 degenerative spondylolisthesis based on her symptoms, neurological findings, and image findings.

The patient underwent a spinous process-splitting laminotomy at L3-4. The L3 spinous process was divided using a bone saw, followed by laminotomy. During ligamentum flavum removal, firm adhesions to the right lateral dura mater were noted and carefully separated, and the ligamentum flavum was resected. No dural tear or cerebrospinal fluid (CSF) leakage was observed intraoperatively. A submuscular closed-hole-type drain was placed under negative pressure, and the wound was subsequently closed. The operative duration was two hours and 18 minutes, with an estimated blood loss of 37 mL. Her lower limb symptoms improved immediately after surgery, and ambulation began on postoperative day 1. There were no postoperative activity restrictions, including toileting, and rehabilitation was initiated the same day.

**Figure 3:**
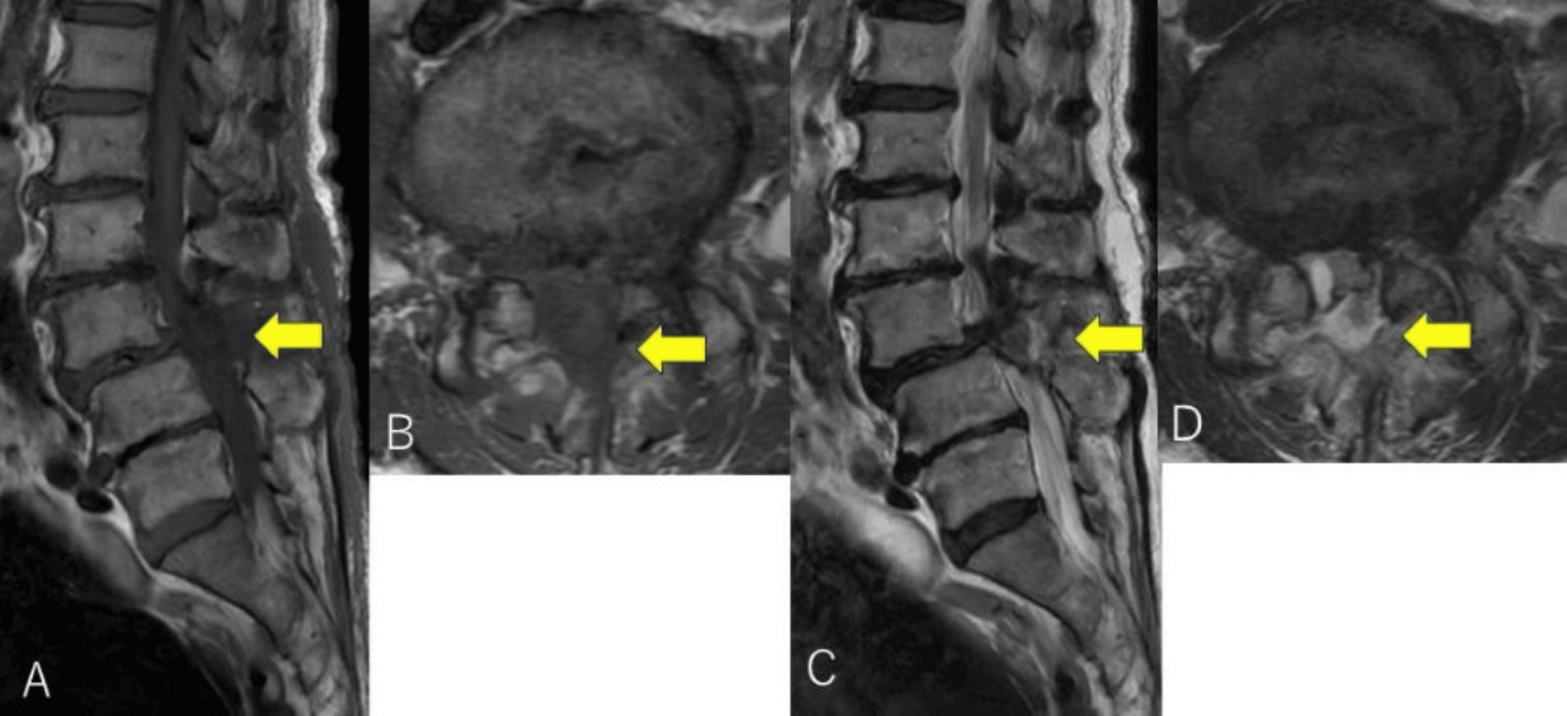
Postoperative lumbar MRI. Lumbar MRI after the initial surgery showing a compressive lesion at the L3-4 decompression site (yellow arrow) with low signal intensity on T1-weighted images (A and B) and  high signal intensity on T2-weighted images (C and D).

The drain was removed on postoperative day 3, with a total drainage volume of 240 mL of serosanguineous fluid. On the same day, the patient developed new-onset perineal numbness after straining during defecation and was found to have urinary retention. Motor function was unchanged, but new sensory dullness in the perineal region was noted. MRI revealed a lesion at the L3-4 decompression site with low T1 and high T2 signal intensity (Figure 3A-3D), indicating a postoperative hematoma causing acute CES.

Emergency reoperation was performed four days after the initial surgery (one day after symptom onset). Upon reopening the incision and re-splitting the L3 spinous process, herniated nerve roots were identified beneath the spinous process. A longitudinal dural tear with a herniated and incarcerated cauda equina was observed at the decompression site. No CSF leakage was noted. The dural tear was extended to release the incarcerated nerve roots, which were repositioned into the dural sac. The dura was repaired with sutures. A new drainage tube was placed on the remaining L3 lamina, and the spinous process was reconstructed before wound closure. No postoperative lower limb paralysis was observed. She regained spontaneous urination and defecation the day after surgery. Postoperative pain and intermittent claudication improved, and the patient was discharged 11 days after the second surgery. At the one-year follow-up, her original lower back and leg pain had not recurred. However, she continued to experience some perineal and lower limb sensory disturbances and remained in pharmacological therapy.

## Discussion

Delayed-onset dural tears that are not identified intraoperatively are rare, with an incidence of approximately 0.2% (two cases per 1,000 spinal surgeries) [1]. Reported risk factors include lumbar procedures (odds ratio (OR) 2.79 compared to cervical spine surgeries), decompression-only procedures (OR 1.72, compared to fusion surgeries), and prolonged operative times exceeding 250 minutes (OR 1.70). Most patients require unplanned reoperation within 1 week due to CSF leak-related symptoms such as headache or pseudomeningocele. Delayed dural tears are associated with increased risks of infection and other complications, underscoring the importance of meticulous intraoperative inspection of the dura [1].

Advanced age and severe lumbar spinal stenosis are also significant risk factors for incidental dural tears [8]. Older patients often have reduced dural elasticity and increased adhesions, which make the dura more susceptible to surgical injury [8].

**Figure 4:**
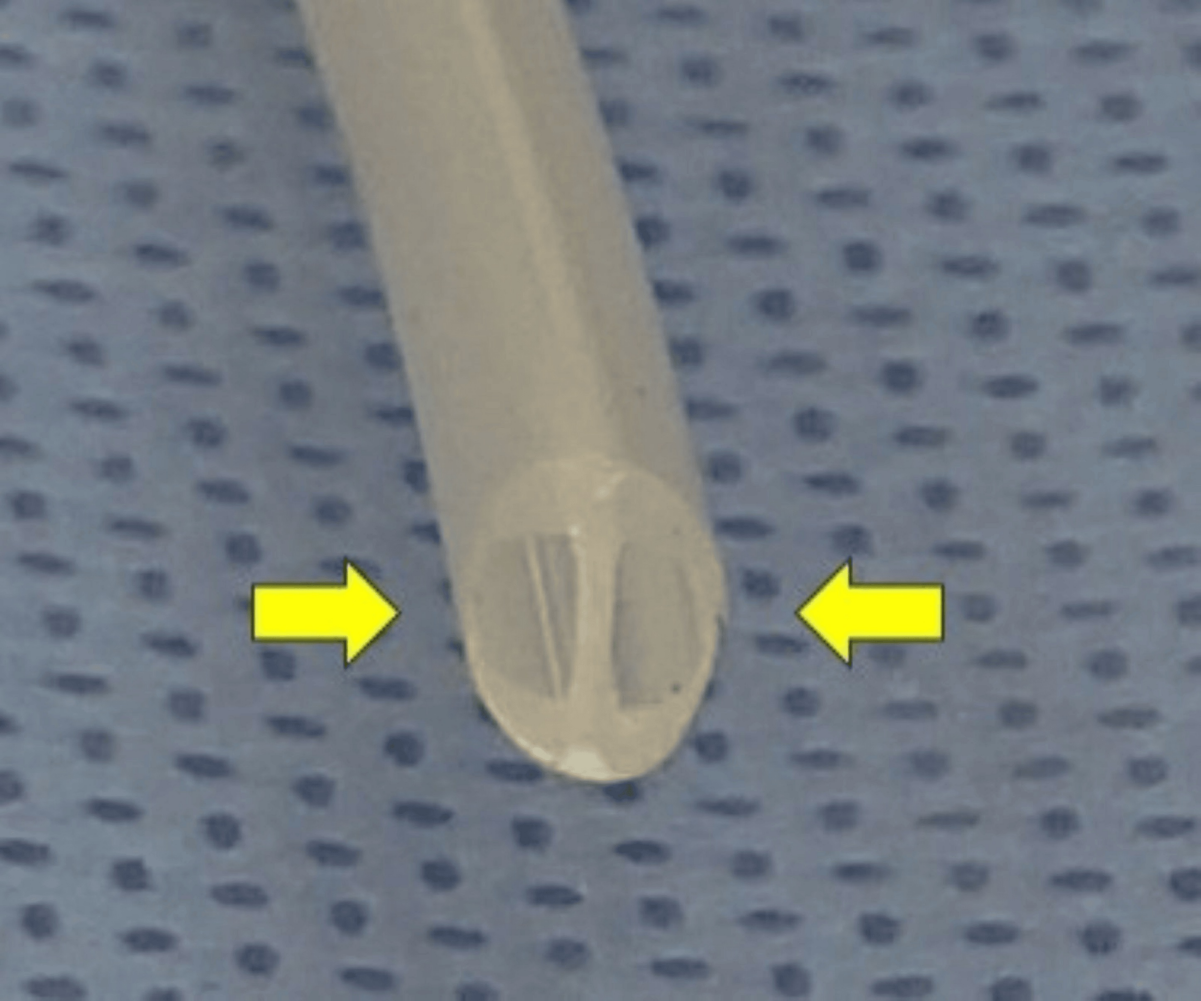
Example of a slit-type drain. Slits are located on both sides, as indicated by the arrows.

Reported mechanisms of delayed dural tears and cauda equina herniation include mechanical trauma from suction drainage, straining, or contact with implants. Suction drains are routinely used in conventional lumbar spine surgery to prevent postoperative hematoma. However, placement of a slit-pore soft drain (Figure 4) near the dura has been reported to cause dural rupture due to the expansion of a small fissure under negative pressure [9]. In our case, the negative pressure generated during drain removal may have caused dural entrapment and tearing at the drain tip. To minimize this risk, the drain tip should be positioned over the lamina rather than adjacent to the dura.

Straining during defecation or coughing (e.g., Valsalva maneuver) can raise intrathecal pressure, potentially enlarging minor dural defects [10]. This may lead to CSF leakage and herniation of nerve roots through the dural defect. Kim et al. reported two such cases of nerve root incarceration [10]. In our case, the patient developed perineal symptoms after straining, which led to herniation and incarceration of the cauda equina through a longitudinal dural tear. Intraoperative Valsalva testing before closure can help detect minor, otherwise occult, dural tears. Furthermore, patient education on avoiding excessive postoperative strain may reduce the risk of delayed dural tears.

In our patient, advanced age, severe stenosis, and likely preexisting dural fragility were contributing factors. Adhesions between the ligamentum flavum and dura may have caused subtle intraoperative injury, which was exacerbated by suction and straining, resulting in delayed rupture and nerve root herniation. The spinal dura mater exhibits anisotropic properties because of its longitudinally aligned collagen fibers. A recent review reported greater tensile strength in the longitudinal compared to the transverse direction, suggesting that tears may preferentially extend along the fiber axis [11]. In our case, the longitudinal tear may indicate that increased pressure, such as during straining, contributed to the dural rupture.

Preventive strategies include gentle handling of the dura, avoiding forceful dissection in areas of adhesion, and ensuring adequate separation between the dura and drains. The drain tip should be carefully positioned over the lamina to avoid direct contact with the dura. The use of non-slit-type drains may also help reduce this risk. At our institution, patients are generally permitted to mobilize even while a drain is in place, and we have not observed any significant complications related to early ambulation in routine practice. However, based on our experience with this case, we acknowledge the possibility that ambulation may contribute to drain tip displacement or fluctuations in negative pressure, potentially increasing the risk of dural injury. Whether bed rest should be recommended while a drain remains in place warrants further investigation.

Diagnosis of delayed dural tears is possible when spinal fluid leakage is observed from the drain. However, diagnosis should be based on a combination of clinical symptoms and imaging findings, particularly when symptoms emerge after drain removal. MRI is useful but not always definitive [6]. Initial management is typically conservative; however, surgical repair is often necessary if symptoms persist for more than three to seven days [10,12] or if complications such as pseudomeningocele, fistula, or infection occur [5] or if cauda equina syndrome is evident. In this case, the patient developed new-onset postoperative cauda equina syndrome. The differential diagnosis of postoperative cauda equina syndrome includes epidural hematoma and abscess, both of which can compress the cauda equina. Based on clinical findings and MRI, an epidural hematoma was initially suspected. However, surgery revealed a dural tear and cauda equina incarceration, both of which were treated successfully. Preoperative suspicion of dural injury was low. Nevertheless, in cases of neurological deterioration following lumbar surgery, delayed dural tears should be considered and addressed promptly.

## Conclusions

We report a rare case of a delayed-onset dural tear with CES following lumbar spinal surgery. In this patient, dural rupture was likely triggered by a straining-induced increase in CSF pressure, exacerbated by preexisting dural fragility, possible minor intraoperative injury, and damage from suction drainage. Postoperative neurological deterioration should raise suspicion for delayed dural injury and prompt timely evaluation and intervention.
